# Nanoneedles Induce Targeted siRNA Silencing of p16 in the Human Corneal Endothelium

**DOI:** 10.1002/advs.202203257

**Published:** 2022-10-17

**Authors:** Eleonora Maurizi, Davide Alessandro Martella, Davide Schiroli, Alessia Merra, Salman Ahmad Mustfa, Graziella Pellegrini, Claudio Macaluso, Ciro Chiappini

**Affiliations:** ^1^ Dentistry Centre Lab University of Parma via Gramsci 14 Parma 43126 Italy; ^2^ Centre for Regenerative Medicine ‘‘S. Ferrari’’ University of Modena and Reggio Emilia Modena 41125 Italy; ^3^ Centre for Craniofacial and Regenerative Biology King's College London London SE1 9RT UK; ^4^ Transfusion Medicine Unit Azienda USL‐IRCCS Reggio Emilia 42122 Italy; ^5^ Holostem Terapie Avanzate S.r.l. Modena 41125 Italy; ^6^ AstraZeneca Granta Park, Great Abington Cambridge CB21 6GH United Kingdom; ^7^ London Centre for Nanotechnology King's College London London WC2R 2LS UK

**Keywords:** gene therapy, nanoneedles, porous silicon, regenerative medicine, siRNA

## Abstract

Nanoneedles can target nucleic acid transfection to primary cells at tissue interfaces with high efficiency and minimal perturbation. The corneal endothelium is an ideal target for nanoneedle‐mediated RNA interference therapy aimed at enhancing its proliferative capacity, necessary for tissue regeneration. This work develops a strategy for siRNA nanoninjection to the human corneal endothelium. Nanoneedles can deliver p16‐targeting siRNA to primary human corneal endothelial cells in vitro without toxicity. The nanoinjection of siRNA induces p16 silencing and increases cell proliferation, as monitored by ki67 expression. Furthermore, siRNA nanoinjection targeting the human corneal endothelium is nontoxic ex vivo, and silences p16 in transfected cells. These data indicate that nanoinjection can support targeted RNA interference therapy for the treatment of endothelial corneal dysfunction.

## Graphical abstract

Nanoneedles deliver topical RNA interference therapy to treat corneal endothelial dysfunction by targeting the proliferative arrest of endothelial cells. Application of nanoneedles loaded with siRNA to the human cornea does not affect its architecture or function, while selectively transfecting endothelial cells to silence the expression of the p16 protein involved in their proliferative arrest.

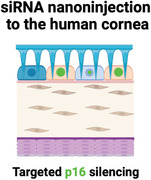

## 1 Introduction

The cornea is the optically clear surface of the eye, essential to overlook the external world by focusing light rays into the eye and allowing vision. Maintaining corneal transparency is necessary to guarantee an optimal eyesight, and this is possible only if all the corneal layers are intact and functional. In particular, the inner monolayer, the corneal endothelium (CE), is fundamental for balancing the liquid exchange that guarantees corneal nourishment, clearance, and transparency. However, the corneal endothelium has a limited regenerative capacity, as human corneal endothelial cells (HCEnCs) are arrested in the G1 phase of the cell cycle.^[^ ^1^ ^]^ Therefore, any loss of HCEnCs is permanent, and progressively leads to an impaired liquid exchange across the cornea, which becomes swollen and opaque, causing loss of vision. The only available treatment for diseases affecting corneal endothelial integrity, such as Fuchs dystrophy, ageing, or iatrogenic damages, is corneal transplantation, which is the most frequent type of graft performed worldwide.^[^ ^2^ ^]^ However, corneal transplantation is an invasive procedure, presenting several limitations related to the risk of allogeneic graft rejection and failure, the need for long‐term immunosuppressive therapy, and the scarce availability of suitable donor corneas.^[^ ^3^ ^]^

Novel approaches for restoring HCEnCs density are key to improving treatment options for corneal endothelial dysfunction. The most appealing and least invasive alternative to corneal transplantation is the regeneration of a patient's own corneal endothelium through transient induction of HCEnCs proliferation either in vivo or ex vivo.^[^ ^4^ ^]^ Moreover, increasing the number of HCEnCs through their expansion in eye bank corneas would be beneficial to reduce tissue wastage,^[^ ^5^ ^]^ as the high cell death rate during storage, in particular following apoptosis in the corneal endothelium, leads to a decrease in HCEnCs density^[^ ^5^ ^]^ and rejection of more than 35% of stored corneas.^[^ ^2^, ^6^ ^]^

A deeper understanding and modulation of the molecular mechanisms regulating HCEnCs proliferation is fundamental to increase their density;^[^ ^7^, ^8^ ^]^ gene therapy has been explored for this purpose in many ways, including by inhibiting apoptosis through overexpression of antiapoptotic genes (e.g., Bcl‐xl),^[^ ^9^, ^10^ ^]^ by inducing cell proliferation through overexpression of transcription factors such (e.g., E2F2),^[^ ^11^ ^]^ or by downregulation of cell cycle inhibitors (e.g., p21, p16,^[^ ^12^, ^13^ ^]^ p27,^[^ ^14^ ^]^ SNAI1, and CDK2).^[^ ^15^ ^]^ Yet, nucleotide delivery to the corneal endothelium is challenging, as the cells are postmitotic and thus hard to transfect.

Similar to the retina,^[^ ^16^ ^]^ the human cornea is an ideal target within the eye for assessing novel gene therapies because of its relative immune privilege that reduces rejection, and easy accessibility that requires minimal surgical manipulation and allows an assisted monitoring. Localized delivery is the preferred administration route of gene therapies to ocular tissues including the corneal endothelium,^[^ ^17^ ^]^ since the systemic route is not efficient, leading to unfavorable bio‐distribution with associated side effects.^[^ ^18^ ^]^

Among local gene delivery approaches for the human cornea,^[^ ^19^ ^]^ viral transduction still raises immunogenicity and safety concerns,^[^ ^20^ ^]^ steering research toward safer nonviral approaches, using lipid based transfection^[^ ^21^ ^]^ and electroporation.^[^ ^22^, ^23^ ^]^ Those nonviral delivery methods can be effective in vitro and ex vivo but still present cell toxicity, do not provide a localized delivery, and do not efficiently address accessibility challenges in vivo.

Nanoneedles^[^ ^24^ ^]^ are a promising approach for corneal delivery, where conventional topical routes are hampered by a drug bioavailability of around 5% that requires large dosing and frequent administration, with risks of severe side effects.^[^ ^25^, ^26^ ^]^ Silicon nanoneedles integrated in tear‐soluble contact lenses are an efficient and painless solution for long‐term delivery of ocular drugs.^[^ ^25^ ^]^ The corneal endothelium is an appealing target for nucleic acid nanoinjection^[^ ^4^ ^]^ and nanoneedles can efficiently transfect siRNA.^[^ ^27^, ^28^, ^29^, ^30^ ^]^ The relative immune privilege of the cornea would reduce the risk of inflammatory response and, since nanoneedles are designed for delivery limited to the superficial layers of a tissue, nanoinjection would selectively reach cells within the corneal endothelial monolayer. Nanoneedle‐mediated delivery, known as nanoinjection, efficiently transfects other postmitotic human cells with high efficiency, without toxicity and with minimal perturbation of cell phenotype.^[^ ^31^, ^32^ ^]^ In particular, porous silicon nanoneedles, entirely biodegradable and capable of hosting large payloads, have emerged as a biocompatible platform that efficiently interfaces with living organisms and human tissue for localized gene therapy and molecular diagnostics, with no off target effects.^[^ ^33^ ^]^ Porous silicon nanoneedles can efficiently load nucleic acids through electrostatic interaction and release their payload progressively over several hours while dissolving.^[^ ^33^ ^]^ Cells uptake such payload through a combination of upregulated endocytosis and direct intracellular presentation.^[^ ^31^, ^34^ ^]^

Here we use porous silicon nanoneedles to develop a nanoinjection approach for RNA interference therapy targeting the human corneal endothelium, aimed at restoring HCEnCs proliferative capacity through p16 (CDKN2A) silencing. In this approach, in vitro nanoinjection of siRNA targeting p16 into primary human corneal endothelial cells preserves their viability and morphological phenotype, while silencing p16 expression, reducing levels of p16 protein, and promoting cell proliferation. Furthermore, nanoinjection targeting the endothelial layer of explanted human corneas preserves cellular structure and does not induce apoptosis while silencing p16 in transfected cells. These results suggest that nanoinjection is a nontoxic method for nucleic acid transfection targeted to the human corneal endothelium.

## 2 Results

### 2.1 Nanoneedle Interfacing with HCEnCs In Vitro

**Figure 1:**
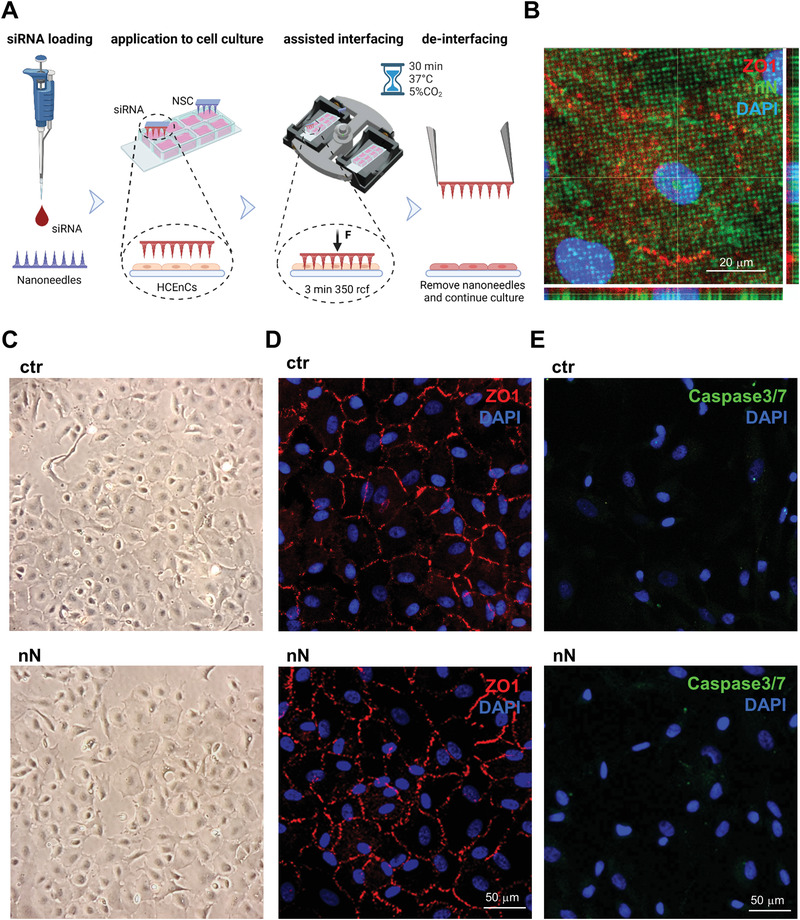
Nanoneedle interfacing with human corneal endothelial cells in vitro. A) Schematic representation of the nanoinjection approach for cultured primary HCEnCs. Image created with Biorender.com. B) Confocal microscopy orthogonal projections of nanoneedles (FITC labeled, green) interfaced with the cytosol, outlined by ZO‐1, and the nucleus of HCEnCs. Nanoneedles colocalize with HCEnCs. ZO‐1 staining (red) with DAPI (blue) nuclear counterstain. Scale bar 20 µm. Images were obtained immediately after nanoneedle assisted interfacing by centrifugation. C) Phase‐contrast image of the primary HCEnCs culture showing retained morphology following nanoneedle interfacing (nN), similar to the untreated control (ctr). Images were obtained immediately after the deinterfacing. D) Immunofluorescence microscopy of HCEnCs showing retained hexagonal morphology and ZO‐1 marker upon nN interfacing (nN) as well as in untreated HCEnCs (ctr). ZO‐1 staining (red) with DAPI (blue) nuclear counterstain. Scale bar 50 µm. Images were obtained 72 h following nanoneedles interfacing. E) Immunofluorescence microscopy of Caspase 3/7 activation. Lack of nuclear staining with faint cytoplasmic staining 72 h following nanoneedle interfacing (nN), comparable to untreated control (ctr) demonstrate lack of Caspase 3/7 activation, indicating absence of apoptotic events. Caspase 3/7 (green) staining with DAPI (blue) nuclear counterstain. Scale bar 50 µm.

We first determined the impact of nanoinjection in primary HCEnCs in vitro. This nanoinjection approach uses a nanoneedle chip loaded with siRNA and placed over the culture, with the nanoneedles facing the cells (**Figure** **1**A). Centrifugation is applied to the system to assist the interfacing. During nanoinjection, confocal microscopy shows multiple nanoneedles colocalizing with the cytosol and nucleus of each cell, indicating successful interfacing (Figure 1B). Comparing treated and untreated cells (ctr) on removal of the nanoneedles 30 min following centrifugation, the cells retained their characteristic morphology in culture (Figure 1C). At 72h following interfacing, the nanoneedle‐treated HCEnCs also showed a native ZO‐1 pattern, indicating a sealed monolayer that preserves the correct morphology (Figure 1D). The expression of ZO‐1 in HCEnCs plasma membranes reveals their characteristic belt of tight junctions, which is strictly connected to their function as a semipermeable barrier, allowing the regulated diffusion of nutrients from the anterior chamber to the whole cornea.^[^ ^35^ ^]^ Lack of Caspase3/7 activation revealed that apoptosis in HCEnCs is entirely absent upon nanoinjection, similarly to untreated cells (Figure 1E).

These data indicate that nanoneedles interfacing with primary HCEnCs in vitro by centrifugation retains cell morphology and ZO‐1 expression and does not induce apoptosis.

### 2.2 Targeted Silencing of p16 in HCEnCs In Vitro

**Figure 2:**
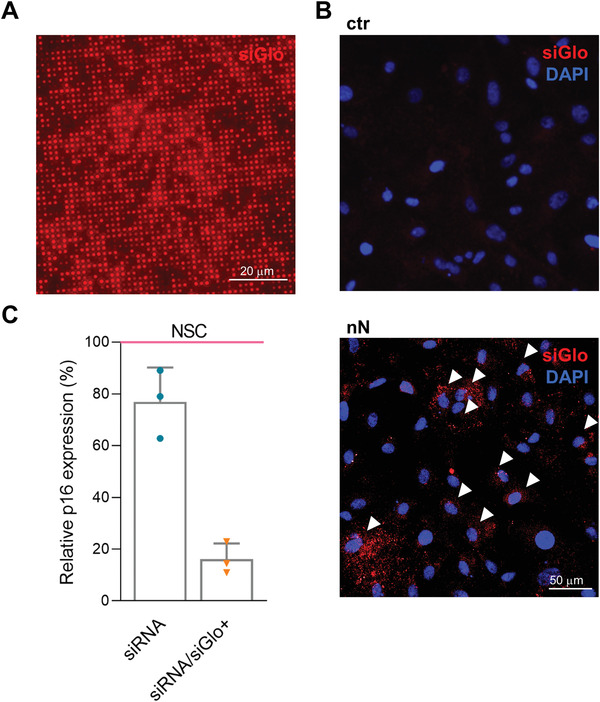
In vitro nanoinjection of p16 (CDKN2A) siRNA in HCEnCs. A) Fluorescence microscopy of nanoneedles loaded with siGlo siRNA. siRNA is adsorbed uniformly across the nanoneedles. Scale bar 20 µm. B) Fluorescence microscopy of HCEnCs 48 h following nanoinjection of siGlo. siRNA accumulates in the cytosol of the cells upon nanoinjection (nN), as compared with the untreated HCEnCs (ctr). White arrows indicate some of the highly transfected cells. siGlo signal (red) with DAPI (blue) nuclear counterstain. Scale bar 50 µm. C) RT‐PCR of p16 expression showing silencing 48 h following p16‐siRNA nanoinjection, normalized and compared to NSC (nonspecific control, pink line). Experiment performed on three primary HCEnCs strains derived from different donors at passage 1 in culture (*n* = 3). The bar on the left (dark blue) indicates overall silencing level, the bar on the right (light blue) is normalized to the fraction of siGlo‐transfected cells in culture. Data are expressed as mean + SD.

We then determined the efficiency of siRNA nanoinjection and its ability to induce targeted gene silencing in vitro. Microscopy analysis revealed thfluorescently‐labeled siGlo Red siRNA abundantly and uniformly loaded onto the nanoneedles prior to interfacing with the cells in culture (**Figure** **2**A). Following nanoinjection, the siGlo was delivered to the cytoplasm of HCEnCs, in 27.6±8% of the treated cells, as quantified from immunofluorescence images (Figure 2B). Three strains of primary HCEnCs derived from different donors were used to assess p16 silencing upon nanoinjection. The delivery of p16‐targeting siRNA to primary HCEnCs resulted in a significant (*p* = 0.04) silencing of the target gene by 23±7% with respect to the nonspecific control (NSC) (Figure 2C). When normalized for the 27.6% transfection efficiency, this approach effectively yielded a 72.4±3.5% silencing of p16 within transfected cells. These results demonstrate the transfection of primary HCEnCs in vitro by siRNA nanoinjection, resulting in significant silencing of the target p16 gene in transfected cells.

### 2.3 Effects of Nanoinjection In Vitro

**Figure 3:**
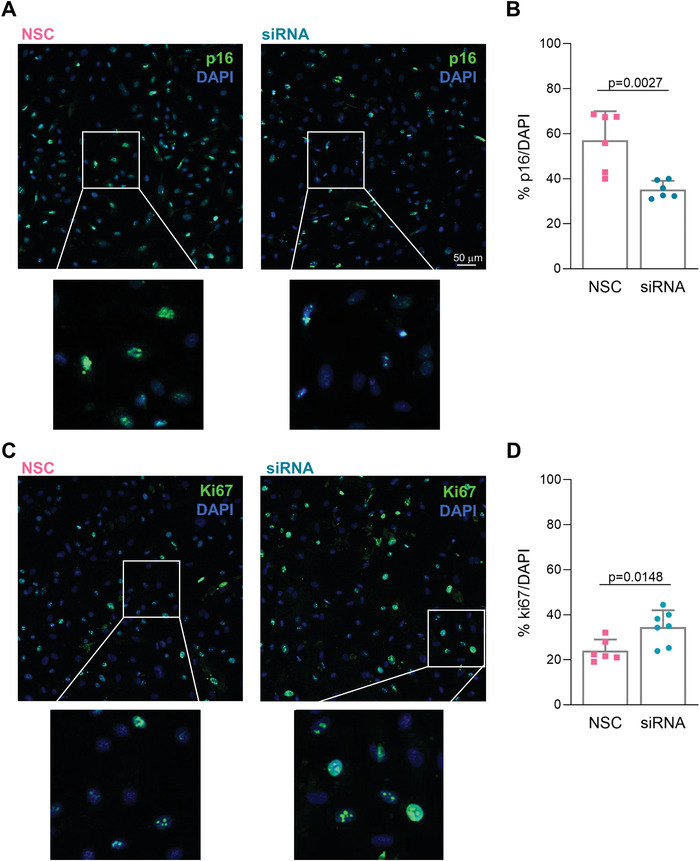
Effects of in vitro nanoinjection. A,B) Nanoinjection with p16‐siRNA (siRNA) induces p16 protein knockdown if compared to NSC nanoinjection (NSC) 72 h following interfacing. A) Immunofluorescence microscopy showing p16 protein expression. p16 staining (green) with DAPI (blue) nuclear counterstain. Scale bar 50 µm. B) Quantification of the fraction of cells expressing p16 for p16‐siRNA (siRNA) and NSC nanoinjected HCEnCs (*n* = 3). p16‐siRNA nanoinjected samples have a statistically significant lower fraction of p16‐positive cells. Data are expressed as mean + SD. Two‐sided *t*‐test was used to assess statistical significance, *p* = 0.0027. C,D) RNAi nanoinjection to HCEnCs enhances their proliferative capacity 72 h following interfacing. C) Immunofluorescence microscopy showing ki67 protein expression in p16‐siRNA (siRNA) and NSC treated HCEnCs in vitro. ki67 staining (green) with DAPI (blue) as nuclear counterstain. D) Quantification of the fraction of cells expressing ki67 protein in p16‐siRNA (siRNA) and NSC treated HCEnCs in vitro (*n* = 3). p16‐siRNA nanoinjected samples have a statistically significant higher fraction of ki67‐positive cells. Data are expressed as mean + SD. Two‐sided *t*‐test was used to assess statistical significance, *p* = 0.0148.

We then evaluated the functional effects of p16 silencing in HCEnCs in vitro. Immunofluorescence quantification of p16‐expressing cells revealed that p16 siRNA nanoinjection downregulated target protein expression when compared with NSC (**Figure** **3**A,B).

The fraction of p16‐expressing cells in vitro was 57±13% for NSC nanoinjection. Indeed, HCEnCs can acquire proliferative capacity^[^ ^4^ ^]^ in culture, which varies based on donor age, tissue state, and cell passage. Although limited, such proliferative capacity yields a fraction of p16 negative cells. Upon p16 siRNA nanoinjection, the fraction of p16 expressing cells was further reduced to 35.2 ± 3.8% (Figure 3B). This reduction is statistically significant (*p* = 0.002) and represents a 21.8% difference in p16 expressing cells, which aligns well with the observed 27% delivery efficiency and the 23% overall p16 silencing (Figure 2). When normalizing the 21.8% reduction in p16 expressing cells for the 27.6% transfection efficiency, this approach effectively yielded a 79% knockdown. These data indicate that the primary human cells, which are transfected through nanoinjection, effectively silence target gene expression and knockdown the synthesis of the target protein.

The reduction of p16 protein expression was supported by a concomitant upregulation of ki67, indicative of an increased proliferation in p16 siRNA‐nanoinjected cells (Figure 3C,D). The fraction of ki67 positive cells following siRNA nanoinjection was 34.4±7.5%, a significant (*p* = 0.014) increase of 10.4% with respect to the 24.0±4.9% observed for NSC nanoinjection (Figure 3D). These data indicate that RNAi nanoinjection therapy has functional outcomes that impact the desired pathway regulation in primary somatic human cells in vitro.

### 2.4 Nanoneedle Interfacing with the Human Corneal Endothelium

**Figure 4:**
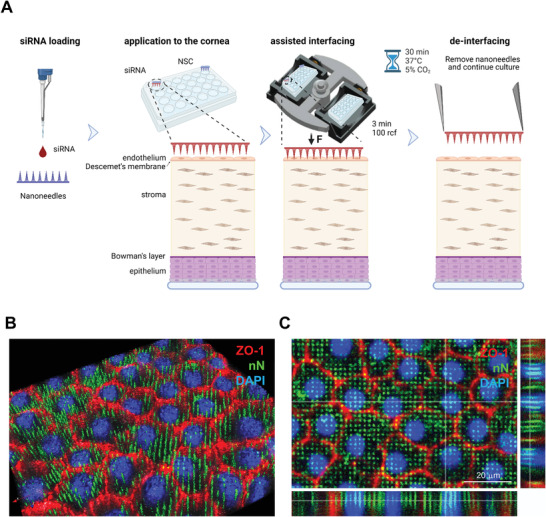
Nanoneedle interfacing with the explanted human corneal endothelium. A) Schematic representation of the nanoinjection approach for explanted human corneas. Image created with Biorender.com. B,C) Immunofluorescence confocal microscopy of the interface between nanoneedles and the endothelium of human cornea explants. Images were obtained immediately after nanoneedle assisted interfacing by centrifugation. Nanoneedles colocalized with HCEnCs and did not protrude beyond them. ZO‐1 (red) localizes in HCEnCs membrane, FITC (green) labels nN and DAPI (blue) nuclear counterstain. B) 3D reconstruction from Z‐stack. C) Orthogonal projections. Scale bar 20 µm.

The successful nanoinjection of HCEnCs in vitro encouraged us to develop a nanoinjection approach for the endothelium in whole corneas (**Figure** **4**). Several delivery studies have exploited ex vivo explanted human corneas with upper sided endothelial surface:^[^ ^36^, ^37^ ^]^ the availability of corneas deemed unsuitable for transplantation and discarded from eye banks^[^ ^2^ ^]^ make those tissues a precious resource for preclinical studies.

The 5 µm thickness of the corneal endothelium monolayer well matches the length of the nanoneedles, ensuring that all HCEnCs can be targeted simultaneously while preventing reaching other cells in the underlying tissues.^[^ ^33^ ^]^ For nanoinjection in explanted corneas, nanoneedle chips loaded with siRNA were placed on the endothelial side of human cornea explants and interfaced with the assistance of centrifugation (Figure 4A). Confocal microscopy shows multiple nanoneedles colocalizing with the cytosol and nucleus of cells throughout the endothelial layer, indicating successful interfacing (Figure S1A, Supporting Information). A 3D reconstruction allowed visualizing the nanoneedle array through, but not beyond, the cells thickness (Figure 4B), which was confirmed by the orthogonal projections (Figure 4C).

**Figure 5:**
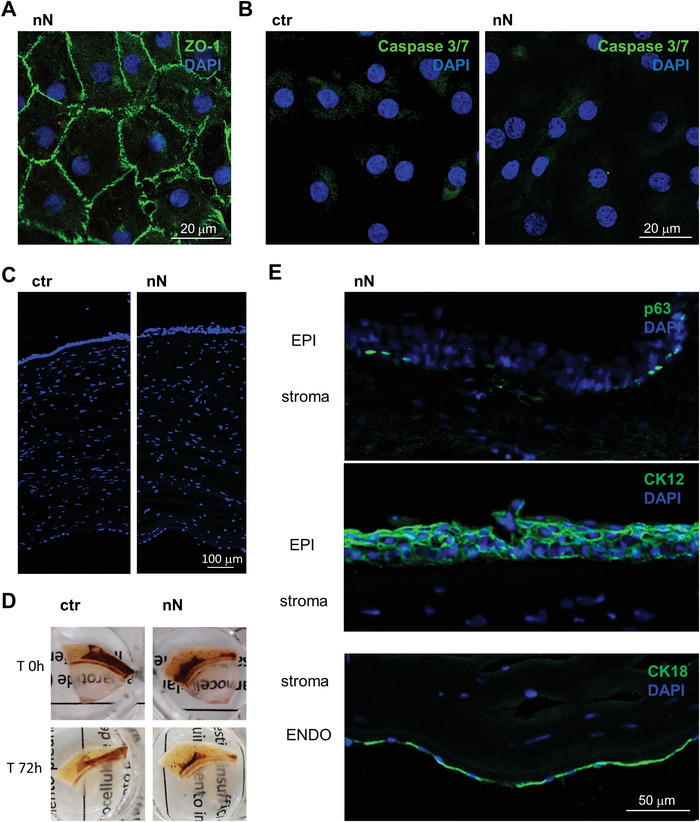
Nanoneedle interfacing preserves cornea integrity. A) Immunofluorescence confocal microscopy image of the explanted corneal endothelium obtained 72 h after nanoneedles interfacing, showing a maintained native endothelial morphology. ZO‐1 (green) staining with DAPI (blue) nuclear counterstain. Scale bar 20 µm. B) Immunofluorescence microscopy of Caspase 3/7 activation 72 h after nanoneedles interfacing. Lack of nuclear staining with faint cytoplasmic staining, comparable to untreated control demonstrate lack of Caspase 3/7 activation, indicating absence of apoptotic events. Caspase 3/7 (green) staining with DAPI (blue) nuclear counterstain. Scale bar 20 µm. C) Fluorescence microscopy of ctr and nN treated corneas (10 µm OCT sections) stained with DAPI (blue) shows that corneal thickness was unchanged in either of the two conditions. Scale bar 100 µm. D) Representative images of explanted corneas before and after (72 h) nanoinjection, showing how corneal clarity is maintained. E) Fluorescence microscopy of nN treated corneal sections, showing the expected expression and localization of epithelial (EPI, p63, and CK12) and endothelial (ENDO, CK18) cell markers (green). DAPI (blue) counterstains nuclei. Scale bar 50 µm.

The cells from explanted corneas retained the expected expression and localization of ZO‐1 at 72 h post‐nanoinjection, indicating that HCEnCs maintained their morphological integrity and ZO‐1 marker expression across the whole endothelial monolayer upon nanoneedle interfacing (**Figure** **5**A). Nanoinjection with FITC labeled nanoneedles revealed that less than 0.4% of the initial nanoneedles were retained within the corneal endothelium following nanoinjection (Figure S1B, Supporting Information). The retained nanoneedles would completely degrade in 72 h, as previously demonstrated.^[^ ^38^ ^]^ Similar to what observed in vitro, nucleic acids nanoinjection also preserved cell viability, as shown by the absence of apoptosis events at 72 h (Figure 5B). Treatment with 10 mm H_2_O_2_ for 2 h was used to induce apoptosis as a positive control (Figure S1C,D, Supporting Information). Corneal thickness (Figure 5C), corneal clarity (Figure 5D) and expression of markers for both the corneal epithelium (p63 and cytokeratin 12, CK12) and endothelium (cytokeratin 18, CK18) (Figure 5E) were maintained in siRNA nanoinjected samples with respect to untreated controls, indicating that nanoneedle treatment is safe for the cornea and does not affect its integrity or marker expression.

DNA loaded within the nanoneedles (Figure S2A, Supporting Information) can be nanoinjected to the cytoplasm of HCEnCs ex vivo (Figure S2B, Supporting Information) and a 3D reconstruction helps visualizing the cytosolic distribution of the nucleic acids following nanoinjection ( Figure S2C, Supporting Information).

These data indicate that the nanoinjection of corneal endothelial cells in human explanted corneas is feasible and nontoxic.

### 2.5 Effects of Nanoinjection to the Human Corneal Endothelium

**Figure 6:**
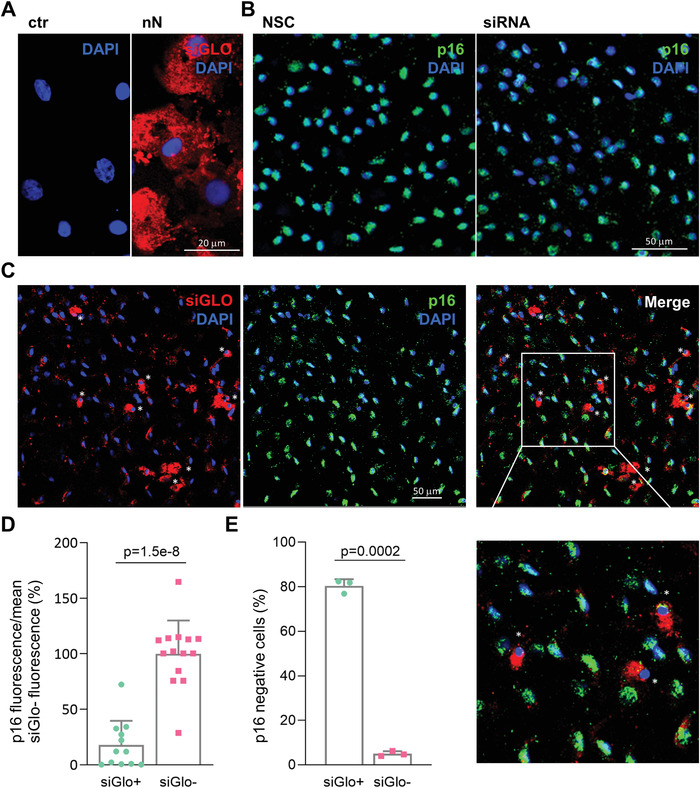
Effects of p16‐siRNA nanoinjection to the explanted human corneal endothelium. A) Immunofluorescence microscopy of siGlo nanoinjection to the endothelium of explanted human corneas. Images were obtained 48 h after nanoinjection. HCEnCs cells display cytosolic siGlO in the area of nanoinjection (nN) as compared to untreated controls (ctr). siGlo signal (red) with DAPI (blue) nuclear counterstain. Scale bar 20 µm. B) Immunofluorescence microscopy of p16 protein expression in NSC and siRNA treated HCEnCs of explanted corneas. p16 (green) staining with DAPI (blue) nuclear counterstain. Scale bar 50 µm. C) Immunofluorescence microscopy of explanted human corneas 72 h following nanoinjection of p16 siRNA (*n* = 3). A significant correlation is visible between siGlo signal and loss of p16 signal, as highlighted by the white asterisks. p16 (green), siGlo (red) staining with DAPI (blue) nuclear counterstain. Scale bar 50 µm. D) Immunofluorescence quantification evaluating the fraction of p16 negative cells in siGlo+ transfected and siGlo‐ untransfected HCEnCs. Values are represented as mean + SD. Two‐sided *t*‐test was used to assess statistical significance, *p* = 1.5e‐8. E) Immunofluorescence quantification of p16 expression levels in siGlo+ transfected and siGlo‐untransfected cells. Values are represented as mean + SD. Two‐sided *t*‐test was used to assess statistical significance, *p* = 0.0002.

We assessed the effects of nanoinjection for RNAi therapy in the endothelium of human corneal explants (Figure 5). A mix of siGlo and p16 siRNA were loaded on the nanoneedles and interfaced with the corneal endothelium. Nanoinjection delivered the siRNA into the cytoplasm of HCEnCs within the endothelial layer as visualized through the siGlo fluorescence (**Figure** **6**A).

Within untreated corneal endothelial layers (Figure S3A, Supporting Information) and NSC treated HCEnCs (Figure 6B) of explanted corneas, cells were uniformly p16 positive (averaged 97.6%±0.08 p16 positive in NSC and untreated corneas, *n* = 4), indicating that nanoneedle interfacing alone does not affect the proliferative block present in the corneal endothelium. Fluorescently labelled siRNA, evaluated before and after nanoinjection, showed that most of the siRNA loaded on the nanoneedles surface was released (Figure S3B, Supporting Information). siRNA nanoinjection ex vivo induced p16 knockdown (Figure 6B–D). In the selected area of interfacing, 10 ± 0.7% of HCEnCs were transfected with siGlo. The p16 signal intensity significantly (*p* = 1.5^−8^) decreased in siGlo transfected (siGlo+, 18 ± 21.5%), as compared to untransfected cells (siGlo‐, 100 ± 30%), confirming that RNA therapy through nanoinjection is effective at silencing p16 (Figure 6D). Overall, 12.2±1.2% of cells showed a downregulation of p16 protein, including 80.4±2.9% of the siGlo‐transfected cells and 5±1.1% of the non siGlo‐transfected cells (Figure 6E). The high‐magnification inserts further show the strong correlation between HCEnCs siGlo transfection (red signal) and p16 protein knockdown (green), as indicated by the white asterisks.

## 3 Discussion

Nanoneedles can efficiently deliver several types of nucleic acids to hard‐to‐transfect cells, among which primary neural, immune and stem cells without appreciably altering their phenotype.^[^ ^31^ ^]^ This study shows that nanoinjection can mediate RNAi in human tissues and provide therapeutically‐relevant outcomes including inducing desired changes in cell function.

Nanoinjection could transfect primary HCEnCs and the endothelium of a human cornea with minimal cellular invasiveness: in vitro or ex vivo nanoinjected HCEnCs maintained cell morphology and ZO‐1 expression; cells appeared functional without signs of apoptosis (Figures 1 and 4), which is an important feature in corneal quality assessment during storage.^[^ ^6^ ^]^ In vitro nanoinjection provided a 72% silencing of p16 mRNA in transfected cells (Figure 2) which knocked down p16 protein expression and induced the desired functional effect of increased HCEnCs proliferation (Figure 3). This enhanced proliferation can be leveraged to increase HCEnCs density, fundamental to regenerate corneal endothelium, avoiding transplantation.

Nanoinjection to explanted corneas showed interfacing throughout the limited thickness of the corneal endothelium (Figure 4A–C and Supp. Figure 2). The p16 knockdown was observed in more than 80% of the siGlo‐transfected HCEnCs (Figure 5), highlighting the potential to further develop nanoinjection for in vivo corneal endothelium reprogramming with minimal mechanical invasiveness. The 80% knockdown in p16 protein expression for the siGlo‐transfected cells matched the 79% p16 protein knockdown within transfected cells observed in vitro (Figure 3).

Nanoinjection provides efficient, nonimmunogenic transfection for different tissues,^[^ ^31^ ^]^ which was confirmed herein for the nonmitogenic corneal endothelium. On the path toward clinical translation, we foresee two approaches to nanoinjection for the treatment of corneal endothelial dysfunction.

First, nanoneedles could be transiently applied to the anterior chamber of the cornea in vivo, adapting existing procedures of corneal endothelial keratoplasty. In endothelial keratoplasty, a donor's corneal endothelial layer is rolled up and introduced into the patient's anterior chamber through a small incision. An air bubble, generated into the anterior chamber, positions the donor's endothelium while regulating eye pressure.^[^ ^39^ ^]^ We foresee a flexible nanoneedle device that could be rolled and introduced into the anterior chamber, using an air bubble to control the positioning and pressure of interfacing with the corneal endothelium, in analogy to the pressure control attained through centrifugation.

Second, nanoneedles applied ex vivo could rescue unsuitable transplant material by regenerating the endothelium of donor corneas prior to implantation, thus improving the quality and reducing the waste of transplant material. In this setup, the rescued corneal endothelial layers can then be transplanted by corneal endothelial keratoplasty.

There are key outstanding developmental steps on the path to the clinical translation of this approach. The optimization of a flexible and curved nanoneedle substrate that precisely adapt to the corneal curve is important to provide conformal interfacing with the tissue. Significant progress toward such devices are being made with the development of nanoneedle for ocular drug delivery.^[^ ^25^ ^]^ Further enhancement to therapeutic efficacy will arise from optimising nanoneedle geometry and chemistry to match cell requirements.^[^ ^25^, ^31^ ^]^ Cell cycle arrest is only one barrier to the recovery of proliferation for cells within the corneal endothelium, with cell–cell inhibitory signaling also playing a major role. Successful therapies will need to address all inhibitory signals in a coordinated fashion. The capacity of nanoneedles to codeliver different therapeutic agents in quantitative ratios will be beneficial in developing these multitherapy strategies.

In conclusion, this study assessed the feasibility of nanoinjection for RNAi therapy in human endothelial corneal cells in culture and in explanted human corneas, demonstrating targeted siRNA transfection, gene silencing, protein knockdown, and functional outcomes.

## 4 Experimental Section

### Fabrication of the Nanoneedles

Porous silicon nanoneedles are fabricated according to the established protocols^[^ ^38^, ^40^ ^]^ over the entire surface of a 100 mm, <100>, p‐type silicon wafer. Nanoneedles are fabricated with < 50 nm tip diameter, 3.5 µm length, 2 × 2 µm^2^ density, and 45% porosity as they previously demonstrated efficient transfection of siRNA and plasmid DNA in live tissue and several cell types in culture.^[^ ^33^, ^34^, ^41^ ^]^ First a 160 nm layer of silicon‐rich silicon nitride is deposited by chemical vapor deposition. The substrate is then patterned by UV photolithography with a square array of 600 nm diameter dots with 2 µm pitch. For the photolithography a 220 nm layer of NR9‐250P photoresist (Futurrex Inc, USA) is spin coated on the substrate with the following parameters 500 RPM/1000 RPMS/5 s, 4000 RPM/5000 RPMS/40 s. The substrate is prebaked at 70 °C for 180 s on a hotplate followed by hard vacuum contact exposure in an MA6 mask aligner (K. Suss GMBH, Germany). The exposed substrate is post‐baked for 60 s at 100 °C on a hotplate, developed in 3:1 RD6:H_2_O developer solution for 12s (Futurrex Inc, USA) rinsed with excess water and dried with N_2_. The photolithographic pattern is transferred into the silicon nitride layer by reactive ion etching (Oxford Instruments, NGP80) with the following parameters: 50 sccm CHF_3_, 5 sccm O_2_, 150 W forward power, 55 mTorr pressure, 150s. The remaining photoresist was stripped with acetone and the substrated cleaned with isopropanol and dried under nitrogen stream. For metal assisted chemical etching (MACE) the native oxide layer was first removed by dipping the substrate in 10% hydrofluoric acid (HF, Honeywell, USA) for 2 min, immediately followed by electroless Ag deposition for 2 min in 100 mL of 20 mm AgNO_3_ (Sigma‐Aldrich) in 10% HF. The substrate was rinsed in water and isopropanol and dried under nitrogen stream. The MACE process formed the porous silicon pillar structures by dipping the substrate in 400 mL of a solution composed of 1 part 30 vol H_2_O_2_ (Sigma‐Aldrich) and 99 parts 10% HF solution. To stop the etch, the wafer was dipped in DI water, then rinsed with excess water and isopropanol, and dried under nitrogen stream. The residual Ag was removed in gold etchant solution (Aldrich) for 10 min. The substrate was rinsed with excess water, isopropanol, and dried under nitrogen stream. The final conical nanoneedle structure was obtained by reactive ion etching in an NGP80 (Oxford Instruments, UK) in the following conditions: 20 sccm SF_6_, 300 W, 100 mTorr for 120s. The 100 mm substrate was diced in 8 × 8 mm chips (Disco, DAD3220) for use in 24‐well plates. Individual chips were oxidized prior to use by oxygen plasma in a Femto plasma asher (Diener, Germany) at 10sccm O_2_, 0.2 mBar, 100 W for 10 min.

Fluorescently‐labeled nanoneedles were obtained by conjugating fluorescein isothiocyanate (FITC) to the oxidized silicon nanoneedles through a silane linker. Aminopropyltriethoxysilane (APTES) was grafted on the silicon surface in 2% APTES ethanoic solution for 2 h. The substrate was then washed 3 times in ethanol and 1 time in DI water. The APTES‐functionalized nanoneedles were reacted in a phosphate‐buffered solution (PBS) of 0.01 mg mL^−1^ FITC for 1 h. The substrate was washed 3 times in PBS and 1 time in DI water, and dried under nitrogen stream until further use.

### Ethical Statement

Human donor corneas, unsuitable for transplantation, were procured by Italian Eye Banks after obtaining written consent from the donor's next of kin for research use. The experimental protocol was approved by ISS‐CNT (Italian National Transplant Centre) and by the local ethical committee (Comitato Etico dell'Area Vasta Emilia Nord, p. 0 002956/20). The tissues were handled in accordance with the declaration of Helsinki.

### Nanoinjection of Human Corneal Endothelial Cells In Vitro

Human corneas, preserved in Eusol at 4 °C, were selected for experiments with the following criteria: age ranging from 4 to 90 years old, no history of corneal diseases, HCEnCs density greater than 1800 cells mm^−2^, death to preservation interval lower than 15 h and used for cultures within 15 days from death (**Table** **1**). The peel and digest method was used to obtain primary culture of HCEnCs. Briefly, intact Descemet's membrane was stripped off the corneas and HCEnCs isolated using 1.5 mg mL^−1^ Collagenase A (Roche, USA) in Dulbecco's Modified Eagle Medium (DMEM, Thermo Fisher Scientific, USA) for 2 h at 37 °C. Isolated HCEnCs were then pelleted at 1200 rpm for 3 min. A further dissociation step with TrypLE (Thermo Fisher Scientific, USA) for 5 min at 37 °C helped cell–cell isolation. After that, the cells were pelleted at 1200 rpm for 3 min and plated in 24 well plates coated with FNC Coating Mix (AthenaES, USA). Dual media method^[^ ^42^ ^]^ was used for expansion of HCEnCs, which were cultured at 37 °C in 5% CO_2_, and the medium was changed every 2 days. HCEnCs between the first and the third passages were employed for experiments: 2 × 10^4^ cells were plated in a chambered 8 well coverslips (IBIDI) 24 h before being treated with nanoneedle chips.

**Table 1:** List of donor human corneas used for the experiments

Cornea n.	Sex	Age	D/P	Endo count	Experiment
1	M	80	21.4	2500	Delivery method optimization
2	M	51	2.1	2700	Delivery method optimization
3	F	81	4.2	2500	Method optimization + Caspase
4	M	84	4	2500	Method optimization + Caspase
5	M	63	12	2600	Method optimization
6	M	90	4.55	2600	Method optimization
7	M	79	13	2700	Method optimization plasmid
8	F	88	2.2	2800	Method optimization plasmid
9	F	60	20	3100	Ex vivo plasmid
10	F	77	7.2	2500	Ex vivo siGlo
11	F	82	17.5	2800	Ex vivo siGlo
12	F	76	8	2300	Ex vivo plasmid
13	F	85			Ex vivo siGlo + plasmid
14	M	79			Ex vivo siGlo + plasmid
15	F	80	10	2217	Ex vivo p16 Ab optimization
16	M	83	5.2	1501	Ex vivo p16 Ab optimization
17	M	78	10	2057	Ex vivo p16 Ab optimization
18	F	72	21	1639	Ex vivo siRNA delivery
19	F	79	23	2283	Ex vivo siRNA delivery
20		81	15.3	2057	Ex vivo siGlo delivery + siRNA KD
21		81	15.3	2222	Ex vivo siGlo delivery + siRNA KD

Cornea n.	Sex	Age	D/P	Endo count	Experiment
22		80	21.5	2192	Ex vivo siGlo delivery + siRNA KD
23		80	21.5	2120	Ex vivo siGlo delivery + siRNA KD
24	M	67	6	1900	In vitro siGlo delivery + siRNA KD
25	F	77	23	1700	In vitro siGlo delivery + siRNA KD
26	M	45	10		In vitro siGlo delivery + siRNA KD
27	F	78	20		In vitro siGlo delivery + siRNA KD
28	M	66	21	1800	In vitro siGlo delivery + siRNA KD
29	F	48	20		Ex vivo interface ZO‐1
30		26	21	3268	In vitro Caspase and interface
31	M	73	9	1800	Ex vivo siRNA delivery
32	F	75	23		Ex vivo siRNA delivery
33		74	5	1900	Ex vivo siRNA delivery

Nanoneedle treatments starts by placing the chips (8 × 8 mm) at the bottom of a 24 well plate, washing them with 2 m HCl to remove impurities and then rinsing twice in distilled water. siRNAs (100 nm) were then loaded onto the chip in a total volume of 10 *µ*L, dissolved in a buffer composed of 0.25 m Glycine and 400 mm KCl, pH 5, and incubated for 30 min. The chip was then applied facing down over the cells monolayer where medium was removed and spun at 350 rcf for 3 min in a swinging bucket centrifuge. Fresh medium was added to the well and the chip was removed after 30 min of incubation.

siRNAs loaded onto nanoneedle chip for the experiments were: siGloRed Transfection Indicator (Dharmacon), Silencer Select Validated siRNA CDKN2A (s218, Thermo Fisher) and Silencer Select Negative Control  (4 390 843, Thermo Fisher), indicated as Non Specific Control (NSC). Plasmid DNA (pm‐mCherry‐N1, Addgene) was labeled with Label It DNA kit (Mirus), following the manufacturer's instructions.

### Nanoinjection of Explanted Human Corneas

Human corneas, preserved in Eusol at 4 °C, were used for experiments within 15 days from explant. Nanoneedle chips (8 × 8 mm) were loaded with siRNAs (100 nm) as described above in the Nanoinjection in vitro section.

Corneal buttons were removed from Eusol, washed in Dulbecco's Phosphate Buffered Saline (DPBS), cut in quarters and each one was located into a well of a 24 well plate with the corneal endothelium facing up. The chip was placed facing down onto the top of the cornea, in direct contact with corneal endothelium, and the plate was then spun at 100 rcf for 3 min in a swinging bucket centrifuge. The chip was left in contact with the cells for further 30 min of incubation with DMEM (Thermo Fisher Scientific, USA), 4% fetal bovine serum (FBS, Fisher Scientific, USA), 4% dextran (Sigma‐Aldrich, USA), penicillin/streptomycin (Euroclone, Italy) at 37 °C and 5% CO_2_ and then placed in a new well, faced up, with fresh medium.

### RT‐PCR

RNeasy plus Micro Kit (Qiagen) was used to extract RNA from HCEnCs, which was then quantified through the Nanodrop 100 (Thermo Fisher Scientific) and reverse transcribed into cDNA with the High Capacity cDNA Reverse Transcription Kit (Thermo Fisher Scientific). RT‐PCR assays were performed using 7900HT Fast Real‐Time PCR System (Thermo Fisher Scientific), choosing the following TaqMan Real Time PCR Assays probes: Human CDKN2A (Hs00923894_m1) and Human GAPDH (Hs02786624_g1). ΔCt and ΔΔCt calculations using GAPDH as housekeeping control were performed to evaluate effective RNA expression. For each condition, all complementary cDNA samples were run in triplicate. Human primary corneal endothelial cultures isolated from three different subjects were used at passage 1 for RT‐PCR analysis.

### Immunofluorescence and Whole Mount Imaging

The following immunofluorescence protocol was used either for in vitro HCEnCs glass slides, whole mount preparations and tissue cryosections.

For cryosections, corneal samples were washed in PBS, embedded in OCT (Killik OCTB, Bio‐Optica, Italy) and stored at −80 °C prior to being cut with the cryostat (Leica, CM1850UV).

Corneal explant samples for whole mount were washed in PBS and treated with sucrose 2 m for 2 min at RT. Immunofluorescence staining, common to all stained samples, was performed by washing all samples in PBS, fixing in 3% paraformaldehyde (PFA) for 15 min at room temperature (RT) and permeabilizing by 0.5% Triton x‐100 (Bio‐Rad, USA) for 10 min. A blocking solution composed of 2% bovine serum albumin (BSA; Sigma‐Aldrich, USA), 2% FBS, and 0.01% Triton in PBS was used to saturate the nonspecific binding sites for 30 min at 37 °C. Primary and secondary antibodies were diluted in blocking solution and incubated for 1 h at 37 °C. Nuclei were counterstained with DAPI (1:40.000 dilution, Roche, USA) for 5 min at RT and mounted with DAKO mounting medium (Agilent, USA). Three rinses in BSA 0.2% were performed between all steps, except before incubation with primary antibody.

In whole mount samples, the corneal slice was finally placed on a glass slide with DAKO mounting medium (Agilent, USA), flattened using a glass coverslip and retained by adhesive tape.

The primary antibodies used were ZO‐1 (1:100, 40‐2200, Thermo Fisher USA), p16 (1:50, ab108349, abcam, USA), ki67 (1:100, ab15580, abcam, USA), while the secondary antibodies were Alexa Fluor 488 antirabbit, 1:2000, and Alexa Fluor 568 antimouse, 1:1000 (Thermo Fisher, USA). Quantification of p16 and ki67 staining was obtained counting the number of positive cells (primary antibody signal), relative to the total number of cells in that field (DAPI staining), expressed in percentage with standard deviation (3 fields for each replicate were collected). p16 fluorescence intensity (Figure 6) was evaluated using ImageJ software and its intensity was normalized to the average of the siGlo‐ group. p16 negative cells were defined by a threshold, determined by measuring the mean fluorescence value in siGlo‐cells calculated in Figure 6D. The value was set at 2 200 000 (Mean Fluorescence x Area) and 3 fields for each condition, containing an average of 50–60 cells, were measured.

Cell apoptosis was evaluated with CellEvent Caspase 3/7 Green (Thermo Fisher, UK), following the manufacturer's instructions. DNA plasmid was labeled with Label IT (Mirus, USA). Reagents used for immunofluorescence are listed in Table S1 (Supporting Information). A confocal microscope (LSM900 Airyscan−Carl Zeiss) was used to obtain the images.

### Statistical Analysis

All graphs show individual data points from all replicates. The bars represent the mean and the error bar the standard deviation for the individual experimental group. Figure 2C is normalized to NSC (nonspecific control); Figures 3B,D and 6E are normalized to total number of cells (DAPI); Figure 6D is normalized to fluorescent intensity of siGlo‐HCEnCs. In vitro experiments presented in Figures 2 and 3 were performed on *n* = 3 strains derived from different donors and on two replicates for each strain. The ex vivo experiments presented in Figure 6 were performed on at least *n* = 3 corneas with at least 4 fields for each cornea evaluated. Two‐sided *t*‐test was used to assess statistical significance as only two groups were compared, and the *p*‐value was reported on the graph. Data analysis was performed on GraphPad Prism 8.4.2 (GraphPad Software Inc., CA).

## Conflict of Interest

The authors declare no conflict of interest.

## Supporting information

Supporting Information

## Data Availability

The data that support the findings of this study are available from the corresponding author upon reasonable request.
