# Supplementary material for: Nanoneedles Induce Targeted siRNA Silencing of p16 in the Human Corneal Endothelium
Source: Adv Sci (Weinh). 2022 Oct 17;9(33):2203257. doi: 10.1002/advs.202203257 (PMC9685449; doi:10.1002/advs.202203257)
Supplement: Supplementary file 1 — Supporting Information [file ADVS-9-2203257-s001.pdf]

ADVANCED SCIENCE
Open Access

Supporting Information

for *Adv. Sci.*, DOI 10.1002/advs.202203257

Nanoneedles Induce Targeted siRNA Silencing of p16 in the Human Corneal Endothelium

Eleonora Maurizi, Davide Alessandro Martella, Davide Schiroli, Alessia Merra, Salman Ahmad Mustfa, Graziella Pellegrini, Claudio Macaluso and Ciro Chiappini**

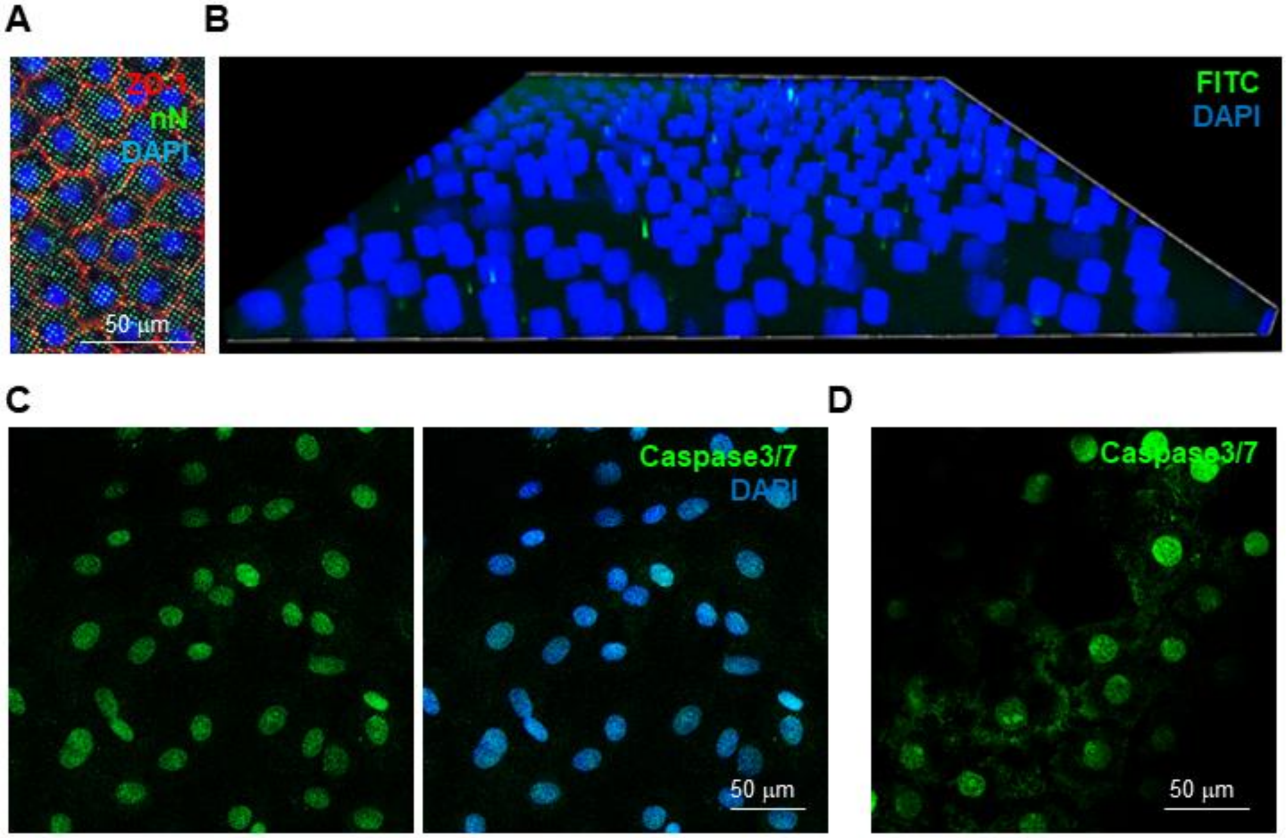

Supplementary Fig1. Evaluation of safety parameters on the explanted corneas. (A) Immunofluorescence overview of a single z-plane across the endothelium of human cornea explants interfacing with nanoneedles (the corresponding 3D reconstruction and orthogonal projection are shown in Figure 4B-C). ZO-1 (red) localizes in HCEnCs membrane, FITC (green) labels nN and DAPI (blue) nuclear counterstain. Scale bar 50 µm. (B) FITC labelled nanoneedles (green) retained into HCEnCs following nanoinjection (3+30 min), represent the 0.4% of the total nN. DAPI counterstains nuclei. (C) Positive control for caspase activation assay in HCEnCs *in vitro.* Strong nuclear green signal indicates Caspase 3/7 activation and induction of apoptosis 2 h following treatment with H_2O_2. DAPI (Blue) nuclear counterstain. Scale bar 50 µm. (D) Positive control for caspase activation assay in explanted human cornea. Strong nuclear green signal indicates Caspase 3/7 activation and induction of apoptosis 2h following treatment with H_2O_2. Scale bar 50 µm.

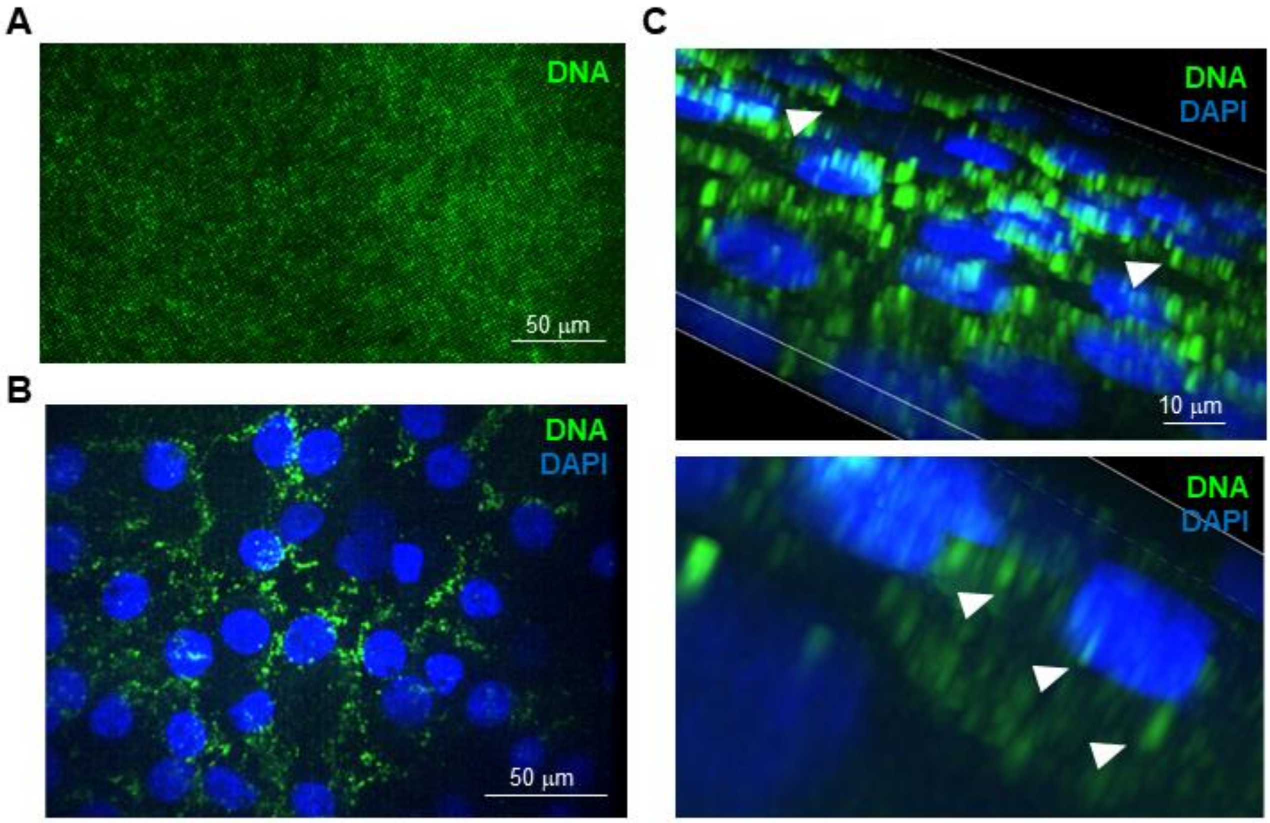

Supplementary Fig2. Nucleic acid delivery to the endothelium of explanted human corneas. (A) Fluorescence microscopy of nanoneedles loaded with an alexafluor-488-labeled DNA plasmid (green) before being applied to the human corneas. Scale bar 50 μm. (B-C) Confocal Fluorescence microscopy of and explanted human cornea 24 h following DNA plasmid (green) nanoinjection, showing cytosolic DNA signal within HCEnCs. DAPI (Blue) nuclear counterstain. (B) z-slice image. Scale bar 50 μm. (C) 3D reconstruction. White arrows indicate cytoplasmic punctate signal within the HCEnCs. Scale bar 10 μm.

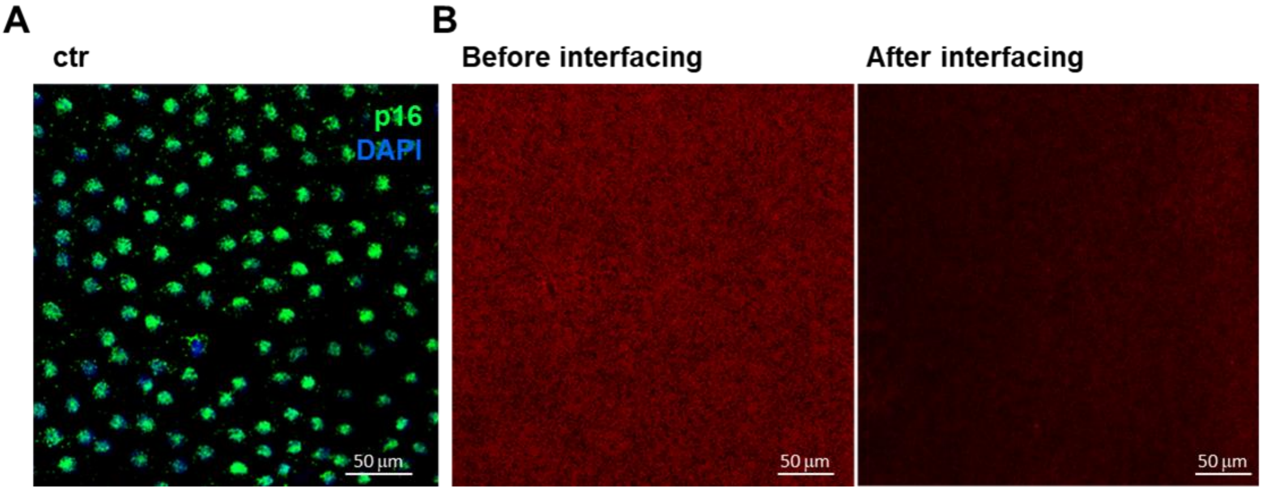

Supplementary Fig3. siRNA nanoinjection to the explanted human corneas. (A) Immunofluorescence microscopy of p16 protein expression in the untreated endothelium of explanted corneas. Nuclear expression of p16 can be detected in almost all HCEnC of the cornea. p16 (green) staining with DAPI (blue) nuclear counterstain. Scale bar 50 μm. (B) Fluorescent images of the needle loaded with fluorescent-labeled siRNA (red) before and after nanoinjection. Scale bar 50 μm.

Assay/protein	reference	dilution
ZO-1	40-2200 (Thermo Fisher)	1:100
p16	ab108349 (Abcam)	1:50
ki67	ab15580 (Abcam)	1:100
CellEvent®Caspase 3/7	C10723 (Thermo Fisher)	1:200
Lable It DNA kit	3225 (Mirus)	

Supplementary Table1. Antibodies and immunofluorescence reagents used for the experiments.
